# Variation in Hypothalamic GnIH Expression and Its Association with *GnRH* and *Kiss1* during Pubertal Progression in Male Rhesus Monkeys (*Macaca mulatta*)

**DOI:** 10.3390/ani12243533

**Published:** 2022-12-14

**Authors:** Hira Zubair, Muhammad Saqib, Muhammad Noman Khan, Shazia Shamas, Shahzad Irfan, Muhammad Shahab

**Affiliations:** 1 Laboratory of Reproductive Neuroendocrinology, Department of Zoology, Faculty of Biological Sciences, Quaid-i-Azam University, Islamabad 45320, Pakistan; 2 Department of Zoology, Rawalpindi Women University, Rawalpindi 46300, Pakistan; 3 Department of Physiology, Government College University, Faisalabad 38000, Pakistan

**Keywords:** GnIH, GnRH, Kisspeptin puberty, rhesus macaque

## Abstract

**Simple Summary:**

An increase in the pulsatile release of gonadotropin-releasing hormone (GnRH) is essential for the onset of puberty. However, the mechanisms controlling pubertal increases in GnRH release are still unclear. In primates the GnRH neurosecretory system is active during the neonatal period but subsequently enters a dormant state in the juvenile period. The present study examined developmental changes in the gonadotropin inhibitory hormone (GnIH) neuronal system, an inhibitory neuropeptidergic system upstream of GnRH neurons, and the relationship among changes that were observed in GnRH and kisspeptin (Kiss1) gene expression during puberty. A significant inverse age-related relationship between GnRH, Kiss1, and GnIH was observed, suggesting GnIH’s potential role in reproductive suppression prior to puberty, with this theoretical ‘brake’ during the pubertal transition. These findings underscore the need for further research on the role that is played by GnIH in reproductive transition to aid in the development of potential GnIH-based drugs to treat pubertal disorders and adult fertility.

**Abstract:**

Modulation of pulsatile gonadotropin-releasing hormone (GnRH) secretion across postnatal development in higher primates is not fully understood. While gonadotropin-inhibitory hormone (GnIH) is reported to suppress reproductive axis activity in birds and rodents, little is known about the developmental trajectory of GnIH expression in rhesus monkeys throughout the pubertal transition. This study was aimed at examining the variation in GnIH immunoreactivity (-ir) and associated changes among GnIH, GnRH, and Kiss1 mRNA expression in the hypothalamus of infant, juvenile, prepubertal, and adult male rhesus monkeys. The brains from rhesus macaques were collected from infancy until adulthood and were examined using immunofluorescence and RT-qPCR. The mean GnIH-ir was found to be significantly higher in prepubertal animals (*p* < 0.01) compared to infants, and significantly reduced in adults (*p* < 0.001). Significantly higher (*p* < 0.001) GnRH and Kiss1 mRNA expression was noted in adults while *GnIH* mRNA expression was the highest at the prepubertal stage (*p* < 0.001). Significant negative correlations were seen between *GnIH*-*GnRH* (*p* < 0.01) and *GnIH*-*Kiss1* (*p* < 0.001) expression. Our findings suggest a role for GnIH in the prepubertal suppression of the reproductive axis, with disinhibition of the adult reproductive axis occurring through decreases in GnIH. This pattern of expression suggests that GnIH may be a viable target for the development of novel therapeutics and contraceptives for humans.

## 1. Introduction

Puberty is defined as the attainment of sexual and somatic maturity [1,2,3]. Puberty enables animals to reproduce and to achieve the adult phenotype [4]. Reproductive axis activity varies during postnatal development in non-human primates [5,6]. More specifically, the reproductive axis remains active during infancy, followed by a transient period of inactivity during juvenile and prepubertal stages [5,6]. At the onset of puberty, an increase in the activity of the reproductive system occurs that continues throughout adult life [7,8]. According to Ojeda, puberty is marked by the reactivation of the hypothalamic gonadotropin-releasing hormone (GnRH) system [3,9]. While a multitude of excitatory (e.g., glutamate) and inhibitory (e.g., gamma amino butyric acid (GABA)), inputs modulate GnRH activity [4,8,10,11], the neuroendocrine regulation of puberty onset is still poorly understood.

Beginning early in this millennium, the discovery of RFamide peptides (peptides having a characteristic Arg-Phe-NH_2_ motif at their C-terminal) [12] with potent actions on the reproductive axis have helped to clarify the mechanisms that contribute to fertility. Among these, kisspeptin is thought to be the most important positive regulator of the reproductive axis, triggering GnRH release during infancy [13,14] and at pubertal onset [15,16,17]. Kisspeptin neurons are primarily localized in the arcuate nucleus region (ARC) of primates with fibers projecting to the mediobasal hypothalamus (MBH), preoptic area (POA), and median eminence (ME) [18]. Kisspeptin-releasing neurons have sex steroid receptors [19], while GnRH neurons do not [20], suggesting that gonadal steroid feedback occurs at kisspeptin cells. GnRH neurons express GPR54, the receptor for kisspeptin [21]. In contrast to kisspeptin, gonadotropin-inhibitory hormone (GnIH) is thought to be the most important negative regulator of the reproductive axis and GnRH secretion. GnIH is a dodecapeptide (SIKPSAYLPLRFamide) that was discovered in the quail brain by Tsutsui and colleagues [22] and subsequently found in other vertebrates from agnathans to humans [23,24,25,26,27,28,29]. GnIH mediates a wide variety of functions including stress, depression, aggression, sleep [30,31,32], and reproduction [33,34]. A multitude of studies has confirmed the suppressive effect of GnIH on the synthesis and release of GnRH and pituitary gonadotropins as well as apoptosis of testicular tissues with seminiferous tubular regression [35,36,37]. Developmental variation in hypothalamic GnIH expression has been reported in various species, including zebrafish [38], Indian major carp [39], European bass [40], cichlids [41], catla [42], European green frogs [43], and mice [44,45]. 

GnIH has been shown to suppress GnRH release through direct actions on the GnRH system and indirectly through alterations of kisspeptin/GPR54 signaling [46,47]. GnIH terminal fiber contacts onto GnRH soma have been reported in rodents, sheep, rhesus monkeys, and humans [23,26,27,48]. However, the developmental pattern of GnIH expression and its association with *GnRH* and *Kiss1* expression has not been investigated in higher primates. Based on findings to date, one can reasonably hypothesize that GnIH might serve as an important prepubertal neurobiological ‘brake’ on the reproductive axis in primates and waning of this neural brake might allow for kisspeptin-dependent/independent GnRH release and the onset of puberty. Therefore, this study examined developmental variation in GnIH expression by immunofluorescence and real time PCR and the relationship between *GnIH-GnRH* and *GnIH-Kiss1* mRNA expression during pubertal development in male rhesus monkeys, a representative higher primate. 

## 2. Materials and Methods

Variation in GnIH expression in male rhesus monkeys from infancy through postnatal development was evaluated via protein and gene expression analyses. Initially, the animals at each developmental stage were characterized according to physical and hormonal parameters, as described previously [16,49]. Hypothalamic blocks of infant, juvenile, prepubertal, and adult animals were collected and processed after euthanizing the animals. Standard single-label immunofluorescence histology was used to stain GnIH nerve terminals and fibers while *GnIH*, *GnRH*, and *Kiss1* mRNA expression was quantified and correlated via RT-qPCR analysis. The association between *GnIH* and *GnRH* and *Kiss1* mRNA was examined to determine whether high levels of GnIH correlated with reproductive axis suppression (i.e., low *GnRH/Kiss1* expression) and low GnIH with reproductive axis competence (i.e., high *GnRH/Kiss1* expression). 

### 2.1. Animals

A total of fifteen intact male rhesus monkeys were employed for the present study. These animals were divided into four different age groups i.e., infant (*n* = 3), juvenile (*n* = 4), prepubertal (*n* = 4), and adult (*n* = 4), according to their body weight, testicular volume, and plasma testosterone levels. The mean ± SEM value of these parameters was as follows: infant: body weight 1.033 ± 0.169 kg, testicular volume 0.12 ± 0.695 mL and testosterone level 0.24 ± 0.0 ng/mL; juvenile: body weight 2.0 ± 0.129 kg, testicular volume 0.326 ± 0.029 mL and testosterone level 0.17 ± 0.0 ng/mL; prepubertal: body weight 4.02 ± 0.131 kg, testicular volume 0.34 ± 0.133 mL and testosterone level 0.22 ± 0.0 ng/mL; and adult: body weight 11.6 ± 1.24 kg, testicular volume 42.4 ± 4.93 mL and testosterone level 2.03 ± 0.4 ng/mL. All the animals were taken captive from Margalla Hills National Park, Islamabad, Pakistan. Each animal was kept in a separate cage under semi-ambient conditions at the Primate Facility of the Department of Zoology, Quaid-i-Azam University, Islamabad, Pakistan. All the animals were fed fresh fruits, peanuts (0900–0930 h), boiled eggs (1100 h), and bread (1300–1330 h). Water was available *ad libitum*. All the experimental procedures were carried out in accordance with the guidelines of the Departmental Committee for Care and Use of Animals (BEC-412). 

### 2.2. Tissue Collection and Processing

Ketamine hydrochloride (Ketamax, Trittau, Germany; 10–20 mg/kg BW, im) was used to deeply sedate the animals prior to brain and testicular tissue collection. Hair was shaved off the head region with a razor, then skin on the skull was thoroughly scrubbed with 70% ethanol and muscle tissue was removed by using a scalpel. After cutting the skull bone in a circular manner with the aid of a sharp bone cutter, the brain was removed from the cranial cavity and immediately placed on a cold glass plate. Hypothalamic blocks, including POA and MBH, were dissected out from the brain as described previously [50]. Briefly, through the mammillary bodies, coronal cuts were made anterior to the optic chiasm. On either side of the midline, a parasagittal cut was made at approximately 4 mm distance. Then, a final horizontal cut was made dorsal to the anterior commissure. Segregated blocks were washed with normal saline. Further hemi-hypothalamic blocks including MBH and POA were made by a cut along the medial line. One hemi-hypothalamic block from all animals was transferred to a fixative (4% paraformaldehyde (PFA)) and was cryopreserved by sequential passage through sucrose solutions to be used in immunocytochemistry. Later, these hemi-hypothalamic blocks were cut into serial sections of 20µm thickness on a cryostat (Bright OTF 5000, A-M systems, Sequim, WA, USA; temperature −25 °C) in the horizontal plane and sections were stored at −20 °C in an anti-freeze solution. The other hemi-hypothalamic block was flash frozen in liquid nitrogen and stored at −80 °C until RNA extraction for RT-qPCR. Single blood samples were collected from all animals in heparinized syringes and their testicular dimensions were noted using Vernier calipers before dissection. For histological purposes, testicular tissue from one testicle of each animal was also collected and was fixed in Bouin’s fixative for 16 h. Testicular tissue was then dehydrated by sequential passage through ascending grades of alcohol, embedded in molten wax, and fixed onto wooden blocks. 

### 2.3. Plasma Testosterone and Testicular Volume Measurement

The concentration of the total testosterone was measured by using a commercially available human enzyme immunoassay (EIA) kit (Astra Biotech GmBH, Luckenwalde, Germany) according to manufacturer’s instructions. Assay sensitivity was 0.05 ng/mL and the inter- and intra-assay coefficient of variation was less than 9% and less than 10%, respectively. At the time of dissection, the testicular volume of all the monkeys was calculated by using the formula V = (πw^2^l)/6, in which ‘w’ denotes width (mm), ‘l’ represents the length (mm) of each testis, and ‘V’ is the volume in ml [51]. The volume of the left and right testis was added to get the total volume of the testes. 

### 2.4. Testicular Morphology

To examine the changes in testicular morphology of monkeys across different ages, the paraffin-embedded tissues were stained with eosin and hematoxylin. For deparaffinization, sections were given two washes in xylol, each for five min. The sections were then rehydrated by passing through descending grades of alcohol (100%, 90%, and 70% each for one minute). Then, the sections were placed in hematoxylin stain for 5 min. Sections were then washed in tap water for 2 min. The sections were then dipped 2–3 times in 1% acid alcohol followed by a 2 min wash with tap water before being placed in eosin for 2 min. After washing with tap water, the sections were dehydrated and given two xylol washes of 1 min each. The sections were then cover-slipped. The epithelial height and tubular diameter were measured and compared among all the monkeys across postnatal development. 

### 2.5. Fluorescence Immunocytochemistry

A total of four sections from each animal were processed using a standard single label immunocytochemistry protocol. Of these 4 sections, 3 were treated with a primary antibody solution while one was used as a primary antibody omitted control section. All the sections were washed in 0.1 M phosphate buffer saline (PBS, pH 7.3; 8 × 15 min each) at room temperature prior to staining. Then, the sections were incubated in a blocking solution containing 10% normal goat serum, 0.05% bovine serum albumin (BSA), and 0.05% Triton-X100 (T-X100) in PBS for one hour at room temperature to block non-specific binding. The sections were then washed with PBS 3 × 15 min. The sections were then incubated with a primary GnIH antibody (rabbit anti-, white-crowned sparrow GnIH antibody (PAC123,124, antigen sequence SIKPFSNLPLRF, generous gift of George Bentley, Berkeley, CA, USA; used at dilution 1:5000) in a buffer solution containing 0.05% BSA and 0.05% TX-100, for 48 h at 4 °C on a shaker, followed by washing for 3 × 15 min. Control sections were incubated without the primary antibody. Later, the sections were incubated in secondary antibody (Cy3-goat anti-rabbit, Cat# 111-165-003; Jackson Immonoresearch Laboratories Inc, West Grove, PA, USA; used at dilution 1:200) solution containing 0.05% BSA and 0.05% TX-100 in PBS for two hours at room temperature, in the dark on a shaker. The control sections were also incubated with the secondary antibody at this stage. Subsequently, sections were washed with PBS 3 × 15 min. After washing, the sections were mounted on super frosted glass slides (CrystalCruz^R^, Cat # Sc-363562; Santa Cruz Biotechnology Inc, Dallas, TX, USA) and left to dry overnight at 4 °C in dark. The next day, the slides were cover-slipped (Microscope Cover Glass, MAS GmbH, Leonberg, Germany, 24 × 50 mm) using anti-fade medium (Immu-Mount^TM^, Cat# 238402, Thermo Shandon Limited, Cheshire, UK). The slides were stored at 4 °C in the dark until fluorescent microscopy was conducted. 

### 2.6. Microscopy

GnIH immunoreactivity was examined by using an Olympus fluorescent microscope (Olympus BX51, Tokyo, Japan) and photographs were taken using a digital camera attached to the microscope. GnIH-ir was visualized using the standard wavelength for Cy-3 (568 nm). The whole MBH area was scanned in three random sections from each animal. The total number of GnIH immunoreactive nerve terminal boutons and fibers in midline hypothalamic regions, especially in the ARC area in each section, was manually counted and the mean ± SEM were calculated for each animal. Testicular sections were viewed under a light microscope and morphological parameters were measured using a micrometer. 

### 2.7. Real Time-Quantitative PCR

#### 2.7.1. Isolation of RNA and cDNA Synthesis

The total RNA was isolated from hemi-hypothalamic block using Wizol^TM^ Reagent (Cat # W76100, Wizbiosolutions, Seongnam, Republic of Korea) according to manufacturer’s instructions. The RNA quantity was measured by using a Thermo Scientific Nanodrop 1000 spectrophotometer (Wilmington, DE, USA). cDNA was synthesized from this RNA using a first strand cDNA synthesis kit (WizScript^TM^, Cat# W2211, Wizbiosolutions) using the supplier’s protocol with the random hexamer primers in a thermocycler (T100 Bio-Rad Thermocycler, Hercules, CA, USA). Briefly, the process involved initial incubation for 5 min at 65 °C, then further incubation at 37 °C for 60 min followed by termination for 10 min at 70 °C. The cDNA samples were placed at −20 °C until further analysis.

#### 2.7.2. Quantitative Real Time PCR

The expression of *GnIH*, *Kiss1,* and *GnRH* genes was evaluated using real-time polymerase chain reaction. The reactions were done by using qPCR Master (SYBR) kit (Wizpure^TM^, Cat # W1401-5, Wizbiosolutions). Each reaction of 10 μL included 5 μL of SYBR Green, 0.45 μL of each primer, 2.5 µL cDNA (1:4 dilution), and 1.6 μL of RNAse-free water. The primers that were used were synthesized by Macrogen company (Seoul, Republic of Korea). The sequences of all the primers that were used with their accession numbers are provided in Table 1. Reaction conditions were pre-denaturation temperature at 95 °C for the 180 s, denaturation temperature 95 °C for 10 s, annealing temperature 60 °C for 15 s, and elongation temperature 72 °C for 20 s. All the reactions were run in duplicate and cycle threshold (Ct) was calculated by using software CFX Maestro software version 2.3 (Biorad, Hercules, CA, USA). Comparative expression was calculated by using the relative Ct method. Every sample was normalized to the endogenous housekeeping gene *GAPDH* expression by using the 2^−ΔΔCT^ method [52,53], taking infant group as calibrator. 

### 2.8. Statistical Analyses

GraphPad Prism Version 8 was used to perform data analysis (GraphPad Software Inc., La Jolla, CA, USA) and the data are expressed as means ± standard error of the mean (SEM). A one-way analysis of variance (ANOVA) followed by Tukey’s multiple comparison post hoc tests, were employed to compare body weights, plasma testosterone levels, testicular morphological parameters, GnIH-ir in the hypothalamus and *GnIH* expression relative to *GnRH* and *Kiss1* at different developmental stages in rhesus monkeys. Pearson’s correlation was used to determine the correlative changes between *GnIH-GnRH* and *GnIH-Kiss1* mRNA expression. Statistical significance was set at *p* ≤ 0.05. 

## 3. Results

### 3.1. Body Weight, Plasma Testosterone, and Testicular Volume

**Figure 1:**
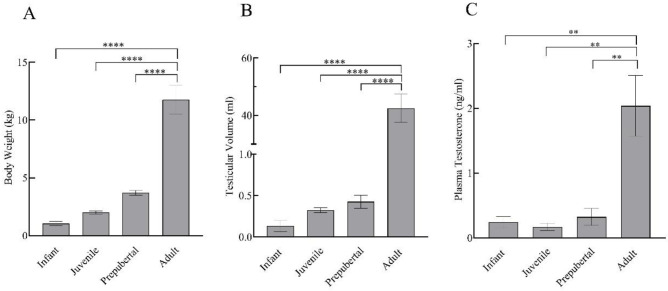
Body weight (**A**), testicular volume (**B**), and plasma testosterone levels (**C**) of infant (*n* = 3), juvenile (*n* = 4), prepubertal (*n* = 4), and adult (*n* = 4) male rhesus monkeys. All data are presented as the mean ± SEM. ** *p* < 0.01, **** *p* < 0.0001.

Body weight and testicular volume showed a prominent increase with the progressing age of the monkeys. Statistically, there was no significant difference (*p* > 0.05) in body weight, testicular volume, and testosterone levels of infant, juvenile, and prepubertal groups, while adults showed significantly higher body weight (F_3,11_ = 50.71; *p* < 0.0001), testicular volume (F_3,11_ = 65.39; *p* < 0.0001), and testosterone levels (F_3,11_ = 11.89; *p* < 0.01) as compared to all the other developmental groups (Figure 1). 

### 3.2. Testicular Morphology 

**Figure 2:**
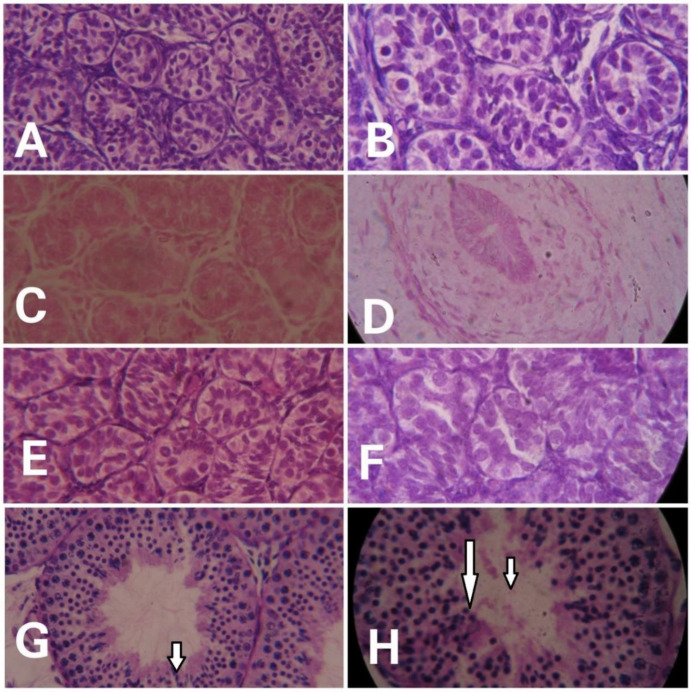
Representative photomicrographs showing the testicular histology of infants ((**A**) = 40×, (**B**) = 100×), juvenile ((**C**) = 40×, (**D**) = 100×), prepubertal ((**E**) = 40, (**F**) = 100×), and adult ((**G**) = 40×, (**H**) = 100×) male rhesus monkeys. Active spermatogenesis (indicated by arrows) and a wide lumen is evident in adult monkeys.

**Figure 3:**
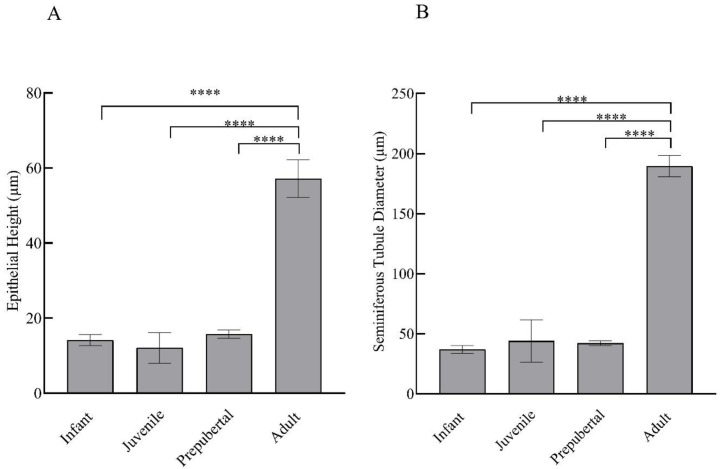
Mean ± SEM epithelial height per tubule (**A**) and seminiferous tubular diameter (**B**) of male rhesus monkeys through postnatal development. **** *p* < 0.0001.

Prominent differences were evident in the histological examinations of hematoxylin- and eosin-stained testicular sections of monkeys in different groups. With advancing age, clear variation in maturation and differentiation of spermatogonia was noticeable. The tubular lumen was closed in infants (Figure 2A,B) whereas juveniles showed a very small luminal space (Figure 2C,D). Monkeys in the prepubertal stage showed a relatively larger lumen (Figure 2E,F) whereas adult monkeys had the maximum luminal space with active spermatogenesis (Figure 2G,H). Epithelial height and tubular diameter showed a prominent increase with age. Adults showed significantly higher (F_3,11_ = 37.82; *p* < 0.0001) epithelial height and seminiferous tubule diameter (F_3,11_ = 49.30; *p* < 0.0001) compared to all the other developmental groups (Figure 3A,B, respectively). 

### 3.3. Developmental Variation in Number of GnIH-ir Terminals Expression Fluorescence 

**Figure 4:**
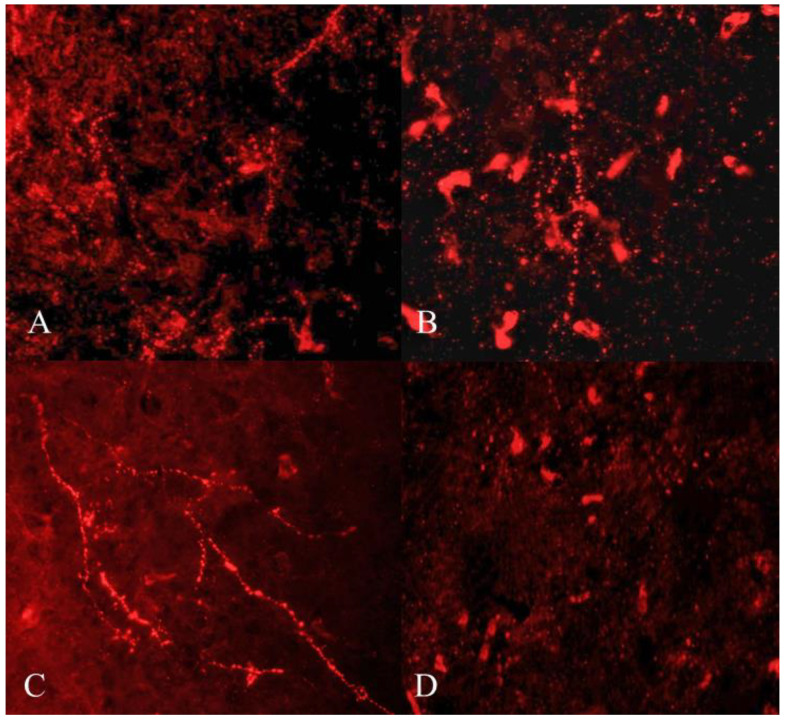
Representative photomicrographs showing GnIH-like immunoreactive terminal boutons and fibers in infant (**A**), juvenile (**B**), prepubertal (**C**), and adult (**D**) male rhesus monkeys.

**Figure 5:**
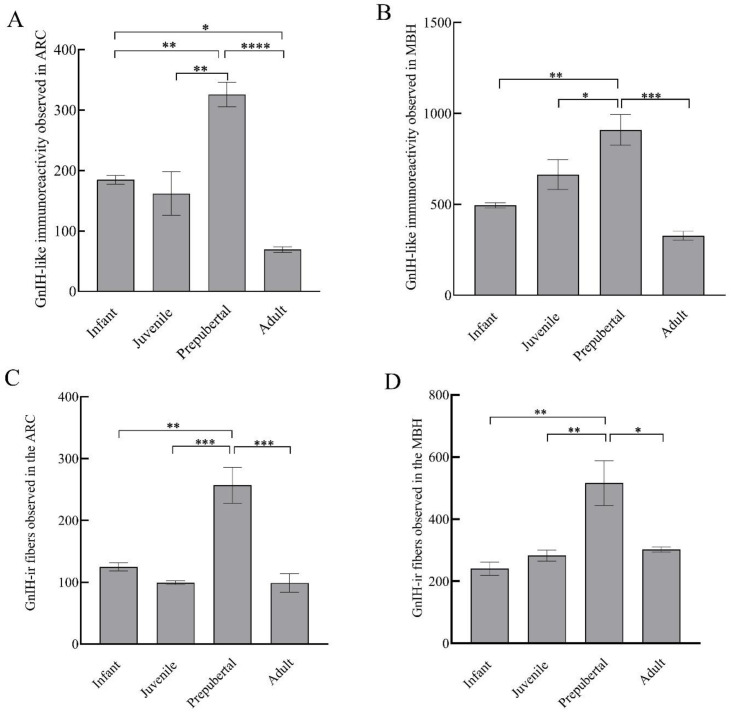
GnIH-like immunoreactive nerve terminals that were observed in arcuate area (**A**), MBH (**B**); and GnIH-like-ir fibers in arcuate area (**C**) and MBH (**D**) of male rhesus monkeys through pubertal development. * *p* < 0.05, ** *p* < 0.01, *** *p* < 0.001, **** *p* < 0.0001.

GnIH-ir terminal boutons were quantified in the ARC and MBH (Figure 4). Significant variation in the number of GnIH-ir nerve terminals in monkey hypothalamus across pubertal development was observed. Specifically in the ARC area, GnIH-ir terminals increased significantly during pubertal development followed by a precipitous decline in adulthood (F_3,11_ = 23.50; *p* < 0.0001). Significantly higher GnIH-ir terminals were observed in prepubertal animals as compared to infants (*p* < 0.01) and juveniles (*p* < 0.001) while significantly reduced expression was seen in adults as compared to prepubertal (*p* < 0.0001) and infant groups (*p* < 0.05) (Figure 5A). The mean number of GnIH-ir terminal boutons in the MBH increased analogously across postnatal development, showing a sharp decline at the adult stage (F_3,11_ = 15.39; *p* < 0.001). Specifically, the number of GnIH-ir terminals was found to be significantly higher in prepubertal animals as compared to infants (*p* < 0.01) and significantly reduced (*p* < 0.001) in adult animals (Figure 5B).

### 3.4. Developmental Variation in Expression of GnIH-ir Fibers 

GnIH-ir fiber expression varied significantly in the ARC region of male monkeys across development (F_3,11_ = 19.09; *p* < 0.001). Specifically, the mean number of GnIH-ir fibers that were expressed in the arcuate area of prepubertal animals was significantly higher than infants (*p* < 0.01) and juveniles (*p* < 0.001), while a significantly reduced number of GnIH-ir fibers was seen in adults as compared to prepubertal (*p* < 0.001) animals (Figure 5C). The expression of GnIH-ir nerve fibers in the MBH varied analogously through pubertal development (F_3,11_ = 9.12; *p* < 0.01). More precisely, the mean number of GnIH-ir fibers was significantly higher in prepubertal animals as compared to infants (*p* < 0.01) and juvenile animals (*p* < 0.01) while staining was found to be significantly reduced in adult animals (*p* < 0.05) as compared to prepubertal animals (Figure 5D). 

### 3.5. RT-qPCR

**Figure 6:**
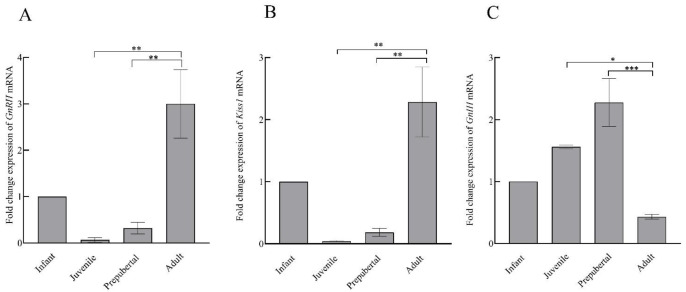
The mean ± SEM hypothalamic fold change expression of *GnRH* (**A**), *Kiss1* (**B**), and *GnIH* (**C**) mRNA during pubertal development in male rhesus monkeys. * *p* < 0.05, ** *p* < 0.01, *** *p* < 0.001.

**Figure 7:**
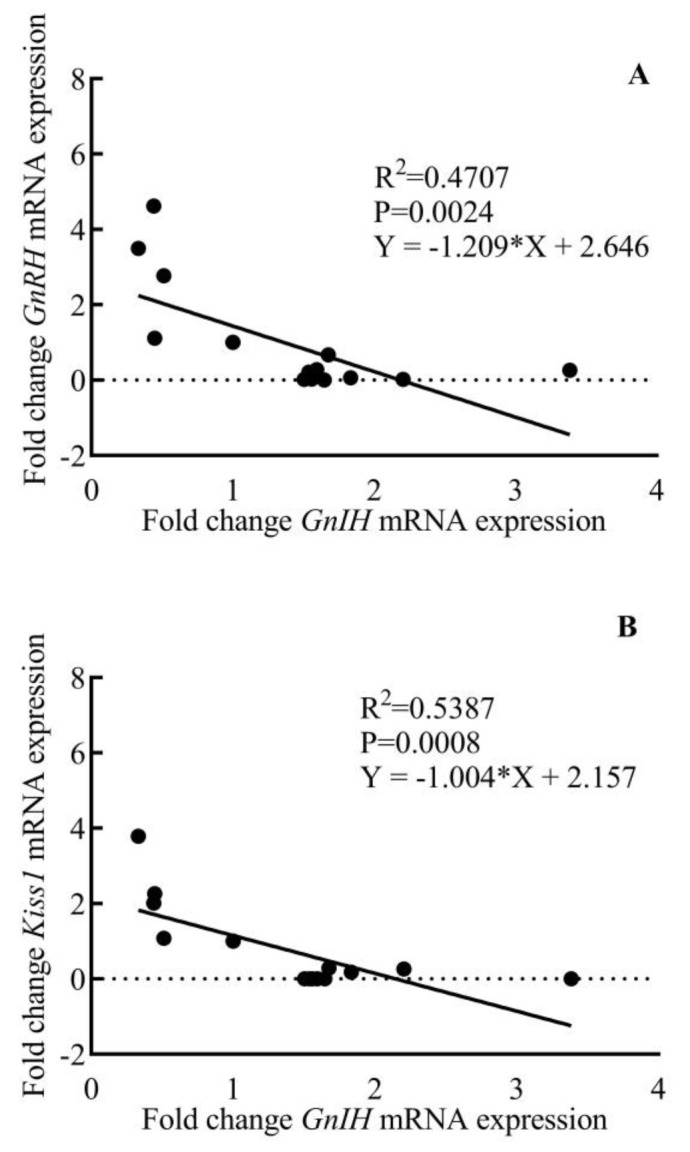
Inverse age-related correlation between hypothalamic fold change *GnRH* (**A**) and *Kiss1* (**B**) expression with *GnIH* expression during pubertal development in male rhesus monkeys.

Comparative changes in the expression of *GnRH, Kiss1,* and *GnIH* mRNA in the hypothalamus of male rhesus monkey during pubertal development are shown in Figure 6. A significant variation was noted in the expression of *GnRH* (F_3,11_ = 11.58; *p* < 0.001), *Kiss1* (F_3,11_ = 12.07; *p* < 0.001), and *GnIH* (F_3,11_ = 14.80; *p* < 0.001) across pubertal development. Significantly higher expression of *GnRH* (*p* < 0.01) and *Kiss1* (*p* < 0.01) in the adult group agree with the active breeding state of the adult animals. *GnIH* expression was found to be significantly higher in prepubertal animals compared to juvenile monkeys (*p* < 0.05) while a sharp decline (*p* < 0.001) in *GnIH* expression was seen in adult animals as compared to prepubertal animals. A significant, inverse correlation was seen between *GnRH-GnIH* (F = 13.34; *p* < 0.01) and *Kiss1-GnIH* (F = 17.52; *p* < 0.001) expression (Figure 7).

## 4. Discussion

In the present study, we examined the pubertal changes in GnIH protein and mRNA expression and its correlation with *GnRH* and *Kiss1* mRNA expression in male rhesus monkeys, a representative higher primate. Our immunofluorescence and qPCR data show a significant increase in the expression of GnIH-ir nerve terminals, fibers, and mRNA during the prepubertal phase of postnatal development as compared to the infantile male rhesus monkeys. In adult animals, we observed a precipitous decline in the number of GnIH-ir neuronal elements. These animals had significantly higher plasma testosterone levels and testicular volume compared to the prepubertal animals, with their testicular histology indicative of a fully active reproductive axis with sufficient spermatogenesis. Together, the present findings suggest that elevated GnIH signaling prior to the onset of puberty causes a hiatus in reproductive axis activity, keeping steroidogenesis and gametogenesis in check, while reduced GnIH signaling in adult animals allows for the onset of puberty by reactivation of the reproductive axis activity. 

In primates, GnRH release is robust during infancy causing steroidogenesis (i.e., sex steroid production) but not gametogenesis (i.e., sperm production) [13,54]. During the juvenile phase of development, GnRH release is dampened, resulting in the relative quiescence of the reproductive axis due to hypogonadotropism [13,54]. At the conclusion of the prepubertal phase of development in primates, pulsatile GnRH secretion in the portal blood resumes, resulting in the release of pituitary gonadotropins, that ultimately act on the gonads. In response to pituitary gonadotropins, the gonads produce sex steroid hormones and gametes, thus activating the reproductive axis, also known as onset of true puberty [14]. Since the discovery of the GnRH neuronal system in the 1970s [55], substantial research has been conducted to decipher the switch that initiates the pulsatile release of GnRH release at puberty [56,57]. Kisspeptin neurons have emerged as an important player in pubertal onset and regulation of gonadal function by maintaining the activity of the neuroendocrine axis during adulthood in primate and non-primate species [58]. As a result, we also quantified *GnRH* and *Kiss1* mRNA expression in male rhesus monkeys of various developmental ages. Our results show higher *GnRH* and *Kiss1* mRNA expression in adult animals compared to juvenile and prepubertal animals. These findings are consistent with previous findings in rodents and primates [15,59,60,61,62]. Likewise, girls with precocious puberty have higher kisspeptin levels compared to prepubertal girls [63], possibly releasing the GnRH pulse generator from the neurobiological brake. 

Despite significant research aimed at determining the neurobiology underlying the developmental regulation of the reproductive axis, the switch that turns GnRH release off in infantile primates has received little attention. After the discovery of GnIH in the year 2000 [22], substantial research has been performed to establish its role in reproductive axis activity in many species [26,27,42,64,65,66]. To completely ascertain the role of GnIH in the conceptual brake on GnRH neuronal activity, we studied the pattern of expression of *GnIH* mRNA during pubertal development and its correlation with *GnRH* and *Kiss1* gene expression. We saw a significant negative correlation between *GnIH-GnRH* and *GnIH-Kiss1* mRNA expression. This finding further strengthens our postulation that higher GnIH signaling during juvenile and prepubertal phases of development keeps the GnRH pulse generator activity in check by downregulating *Kiss1* expression in the hypothalamus, while a decrease in GnIH signaling at the end of the prepubertal phase brings about the resumption of the GnRH pulse generation, thus activating the reproductive axis. 

GnIH-ir nerve terminals and fibers were seen in midline hypothalamic nuclei (ARC) where GnRH neurons are located, suggesting possible innervation by GnIH projections, a finding that is consistent with previous findings in other species [23,25] and in higher primates [26,27]. A subtle variation in the expression of GnIH neuronal elements was noticed with advancing age in rhesus monkeys. Our results are in line with previous findings in mice where GnIH-ir was found to be significantly decreased in pubertal animals compared to prepubertal animals [44,45]. This finding suggests that GnIH may directly inhibit GnRH neuronal signaling or might do so via intermediary neuronal systems that are present in these brain areas such as the kisspeptin neuronal population in the ARC region. Previously, it was established that centrally administered GnIH interferes with the pituitary gonadotropins release in white-crowned sparrows [67], Syrian hamsters [23], and rats [36] and reduces the firing activity of the GnRH neurons [68,69]. Thus, variation in GnIH expression during pubertal development might directly modulate the GnRH pulse generator activity controlling the activation of the reproductive axis. However, ascertaining morphological interactions between GnIH with GnRH and kisspeptin neuronal elements will help in understanding the role of GnIH signaling during sexual maturation of higher primates. It is also highly plausible that GnIH regulates GnRH pulse generation activity of an individual based on energetic state via an interaction with energy-sensitive POMC neurons in the ARC [70,71]. GnIH might regulate other physiological functions by modulating the activity of other neuronal systems that are present in MBH nuclei, including the regulation of prolactin release via dopamine neurons that are present in ARC [72]. Finally, GnIH may downregulate the reproductive axis activity under stressful conditions by modulating the activity of the hypothalamic pituitary adrenal axis by directly affecting corticotropin-releasing hormone (CRH) secretion from the PVN [73]. 

Although there is a clear trend of age related changes in GnIH expression, our sample size is small. Smaller sample sizes are common in studies of higher primates compared to studies in rodents because of ethical considerations, especially the studies that involve euthanizing the animals for tissue collection and where animals cannot be repeatedly examined [74,75,76,77,78,79]. Also, although hypothalamic sections were carefully selected, only a limited number of sections were scanned. Examination of the whole hypothalamus may have provided a more detailed view of the modulation in GnIH signaling with advancing age in various brain nuclei. The ages of the animals cannot be determined with certainty as all the animals were captured from wild. Although body weight, testicular morphology, and plasma testosterone measures, together allow a relatively accurate assessment of age by comparison to previous work [16,49,80]. It is possible that we did not observe differences from infant to prepubertal animals due to age variability in these groups. Future studies involving confocal analysis of the colocalization and interactions of GnIH with GnRH and kisspeptin and other neuropeptides may provide a more detailed assessment of the mechanisms that are responsible for the neuroendocrine regulation of puberty onset in higher primates. 

## 5. Conclusions

In summary, the findings of the present study suggest that suppression of reproductive axis activity during the juvenile and prepubertal phase of development in higher primates is associated with an increase in GnIH tone as indicated by an increase in the GnIH peptide and gene expression at these stages. Furthermore, the negative correlation of *GnIH* expression with *GnRH* and *Kiss1* expression that was observed in this study implies that GnIH might serve as an important player in the neurobiological brake on reproductive axis activity by decreasing kisspeptin and GnRH activity directly, or via intermediary neuronal systems. Based on the current findings, it can also be suggested that, in addition to reproductive axis activity, GnIH might also regulate other neuronal populations to influence motivated behaviors, including reproductive behavior. The present findings set the stage for future studies examining whether GnIH directly inhibits GnRH pulse generation by inhibiting GnRH neuronal activity or by intermediary neural pathways that are present in the DMH, ARC, POA, and PVN. Further, advanced genomic and pharmacological studies will advance understanding the role of this neuropeptide in pubertal development and sexual differentiation and guide the development of novel therapeutic approaches in the treatment of hormone-dependent diseases such as precocious puberty, endometriosis, uterine fibroids, benign prostatic hyperplasia, and prostatic and breast cancers. Human GnIH may also have potential as a novel contraceptive and in the treatment of fertility related disorders. 

## Figures and Tables

**Table 1:** Sequence, accession number, and product length of the primers that were used for RT-qPCR.

Gene	Accession#	Primer Sequence (5′ to 3′)	Product Length
***GnRH***	S- 75918	Rev: TTTCCAGAGCTCCTTTCAGG	134
		For: AGATGCCGAAAATTTGATGG	
***Kiss1***	XM-028852143.1	Rev: TGACTCCTCTGGGGTCTGAA	141
		For: GGACCTGCCGAACTACAACT	
***GnIH***	NM-001033115.2	Rev: ATTGGCACATGGTGAATGC	118
		For: CCTCGTGAGACGGGTTCTTA	
***GAPDH***	NM-001195426.1	Rev: TTGATGACGAGCTTCCCGTT	119
		For: TGTTGCCATCAATGACCCCT	

## Data Availability

Not Applicable.
